# The prosurvival role of autophagy in Resveratrol-induced cytotoxicity in human U251 glioma cells

**DOI:** 10.1186/1471-2407-9-215

**Published:** 2009-06-30

**Authors:** Jun Li, Zhenghong Qin, Zhongqin Liang

**Affiliations:** 1 Department of Pharmacology, Soochow University School of Medicine, Suzhou, PR China; 2 Laboratory of Aging and Nervous Diseases Soochow University School of Medicine, Suzhou, PR China

## Abstract

**Background:**

Previous study reported that resveratrol has anti-tumor activity. In this study, we investigated the involvement of autophagy in the resveratrol-induced apoptotic death of human U251 glioma cells.

**Methods:**

The growth inhibition of U251 cells induced by resveratrol was assessed with methyl thiazolyl tetrazolium (MTT). The activation of autophagy and proapoptotic effect were characterized by monodansylcadaverine labeling and Hoechst stain, respectively. Mitochondrialtransmembrane potential (ΔΨm) was measured as a function of drug treatment using 5,5',6,6'-tetrachloro-1,1',3,3'-tetraethylbenzimidazolylcarbocyanine iodide (JC-1). The role of autophagy and apoptosis in the resveratrol-induced death of U251 cells was assessed using autophagic and caspase inhibitors. Immunofluorescence, flow cytometry, and Western blot analysis were used to study the apoptotic and autophagic mechanisms.

**Results:**

Methyl thiazolyl tetrazolium (MTT) assays indicated that resveratrol decreased the viability of U251 cells in a dose- and time-dependent manner. Flow cytometry analysis indicated that resveratrol increased cell population at sub-G1 phase, an index of apoptosis. Furthermore, resveratrol-induced cell death was associated with a collapse of the mitochondrial membrane potential. The pan-caspase inhibitor Z-VAD-fmk suppressed resveratrol-induced U251 cell death. Resveratrol stimulated autophagy was evidenced by punctuate monodansylcadaverine(MDC) staining and microtubule-associated protein light chain 3 (LC3) immunoreactivty. Resveratrol also increased protein levels of beclin 1 and membrane form LC3 (LC3-II). Autophagy inhibitors 3-methylademine (3-MA) and bafilomycin A1 sensitized the cytotoxicity of resveratrol.

**Conclusion:**

Together, these findings indicate that resveratrol induces autophagy in human U251 glioma cells and autophagy suppressed resveratrol-induced apoptosis. This study thus suggests that autophagy inhibitors can increase the cytotoxicity of resveratrol to glioma cells.

## Background

Autophagy is a degradative process involving sequestration of cytoplasm and organelles into double-membrane vesicles that traffic the contents to lysosomes where recycling takes place [1-3]. It is a genetically programmed, evolutionarily conserved process, typically observed in hepatocytes after amino acid deprivation [4]. Recently, extensive attention has been paid to the role of autophagy in cancer development and therapy [5-8]. There is increasing evidence suggesting that radiation and chemotherapeutic agents induce autophagy in many human cancer cell lines [9-11]. In some cases, autophagy is one of the defensive mechanisms in cancer cells [11-14]. By inducing autophagy, cancer cells recycle molecules for biosynthetic or metabolic reactions and subsequently tailoring themselves to adverse conditions after anticancer therapy. On the other hand, persistent activation of autophagy can also lead to programmed cell death [15,16]. This type of autophagy is irreversible and is termed as type II programmed cell death or autophagic cell death, in contrast to apoptosis, which is referred to type I programmed cell death [12,17]. The mechanisms by which autophagy differentially affects tumor cell survival remain to be uncovered.

Malignant gliomas are the most common primary brain tumors in the central nervous system. These tumors increasingly grow and invade into the surrounding brain parenchyma. Despite advances in surgical preventations and treatments, the prognosis of this disease remains poor [18]. Therefore, developing novel strategies are essential in order to improve effectiveness of treatments for this disease.

In recent years, many compounds that are contained in the diet and beverages have been identified as potential chemopreventive agents. Among them is resveratrol (Res), a natural product highly enriched in grapes, peanuts, red wine, and a wide variety of food sources [19]. Its exact physiological function is still not known, but it has attracted research attention, owing to its cardioprotective, antioxidant, anti-inflammatory activities and cancer chemopreventive properties [20,21]. Res has a number of biological effects in a variety of cell culture systems: it produces variable anti-tumor effects in different tumor cell lines [19]. Res has been shown to have growth-inhibitory activity, and induces apoptotic cell death in a number of human cancer cell lines as well as in animal models of carcinogenesis. In U251 glioma cells, treatment with Res led to growth inhibition, induction of apoptosis and G0/G1-phase cell cycle arrest [22]. Res also showed antiproliferative activity in JB6 mouse epidermal, CaCo-2 colorectal and A431 epidermoid carcinoma cell lines [23-25]. Its effects in ovarian cancer cell lines are more complicated. Res can induce ovarian cancer death through two distinct pathways: apoptosis and autophagy [26]. In the mouse skin carcinogenesis model, Res inhibited the three major steps of carcinogenesis: initiation, promotion, and progression [19]. In human retinoblastoma cells, Res inhibits cell proliferation and stimulates apoptosis through activation of the mitochondrial apoptotic pathway [27]. Thus, multiple mechanisms may be activated by Res, depending on the specific cell types and cellular environment. However, the precise role of autophagy in Res's antitumor effects requires further investigation.

## Methods

### Chemicals

Res was purchased from Sigma Chemical Co. (St. Louis, MO, USA) and dissolved in DMSO as a stock solution of 100 mmol/L. The pan-caspase inhibitor Z-VAD-fmk was obtained from Promega. RPMI-1640 was obtained from Gibco (Rockville, MD, USA) and fetal bovine serum was purchased from Hangzhou Sijiqing Biological Engineering Materials (Hangzhou, China).

### Cell Culture

Human glioma U251 cells were purchased from the Shanghai Institute of Cell Biology, Chinese Academy of Sciences (Shanghai, China). The cells were maintained in RPMI-1640 medium supplemented with 10% heat-inactivated fetal bovine serum and 0.03% L-glutamine (Sigma, St Louis, MO, USA) and incubated in a humidified 5% CO_2 _incubator at 37°C. The cells in the mid-log phase were used in the experiments.

### Cell Viability Assay

Cell viability was assessed by MTT assay as described previously [28]. Cells were plated in 96-well plates at the density of 10,000 cells in 100 μL medium per well one day before the experiment. MTT solution was added to the culture medium (500 μg/ml final concentration) 4 h before the end of treatments and the reaction was stopped by addition of 10% acidified SDS (100 μl) to the cell culture. The absorbance value (A) at 570 nm was read using an automatic multiwell spectrophotometer (Bio-Rad, Richmond CA, USA). The percentage of cell death (growth inhibition) was calculated as follows: cell death(%) = (1-A of experiment well/A of positive control well) ×100%.

### Assessment of Mitochondrial Membrane Potential

To measure the mitochondrial membrane potential (ΔΨm), 5,5',6,6'-tetrachloro-1,1',3,3'-tetraethylbenzimidazolylcarbocyanine iodide (JC-1), a sensitive fluorescent probe for ΔΨm was used [29]. The U251 cells were cultured in 24-well plates for 24 h, and then exposed to the Res (150 μM) for various times. Cells were then rinsed with PBS twice, stained with 1 mL 10% RPMI-1640 medium containing 5 μmol/L JC-1 (Molecular Probes, USA) for 30 min at 37°C. Cells were rinsed with ice-cold PBS twice, resuspended in 1 mL ice-cooled PBS, and instantly assessed for red and green fluorescence with flow cytometry (EPICS-XL, Beckman, USA) [30,31]. A 488 nm filter was used for the excitation of JC-1. Emission filters of 535 and 595 nm were used to quantify the population of mitochondria with green (JC-1 monomers) and red(JC-1 aggregates) fluorescence, respectively [30]. Frequency plots were prepared for FL1 (green) and FL2 (red) to determine the percentage of the mitochondria stained green (low membrane potential) and red (normal membrane potential).

### Cell cycle analysis

PI staining was used to analyze DNA content. U251 cells were plated in 10-cm culture dishes at density determined to yield 60–70% confluence within 24 h. Cells were then treated, with or without Res 150 μM for 24–48 h, were trypsinized, stained with PI by the Cellular DNA Flow Cytometric Analysis Reagent Set (Boehringer Mannheim, Indianapolis, IN, USA), and analyzed for DNA content using the FACScan (Becton Dickinson, San Jose, CA, USA) as previously described [32]. Data were analyzed by Cell Quest software (Becton Dickinson). All experiments were performed in duplicate and yielded similar results.

### Apoptosis detection assay

To detect apoptosis morphologically, we treated U251 cells with Res for 72 h, fixed them with 4% paraformaldehyde, and stained them with Hoechst 33258(0.5 μg/ml) for 15 min.

### Labeling of Autophagic Vacuoles with monodansylcadaverine (MDC)

The autofluorescent agent MDC (Sigma) was recently introduced as a specific autophagolysosome marker to analyze the autophagic process [33]. U251 cells were treated with Res 150 μM for 3–24 h. Autophagic vacuoles were labeled with MDC by incubating cells with 0.05 mM MDC in RPMI1640 at 37°C for 10 min at various times after Res treatment. After incubation, cells were washed three times with PBS and immediately analyzed with a fluorescence microscope (Nikon Eclipse TE 300, Japan) equipped with a filter system (V-2A excitation filter:380/420 nm, barrier filter:450 nm) [34]. Images were captured with a CCD camera and imported into Adobe Photoshop.

### Immunofluorescence of microtubule-associated protein LC3

LC3, a mammalian homologue of Apg8p/Aut7p essential for amino acid starvation-induced autophagy in yeast [35], was recruited to the autophagosome membranes in the Apg5-dependent manner [36]. Therefore, autophagosome membrane association with LC3 is a specific marker for autophagy [33]. After being incubated with Res 150 μM for 3–24 h, cells were washed with PBS and then fixed with paraformaldehyde (4% w:v). After rinsing in PBS, the cells were blocked with 0.1% Triton X-100 containing 1% bovine serum albumin in PBS for 1 h. This was followed by incubation in goat polyclonal antibody against microtubule-associated protein 1 light chain 3 (LC3; 1:100, sc-16756; Santa Cruz Biotechnology, Santa Cruz, CA, USA) for 24 h at 4°C in a humidified chamber. After 3 washes in PBS, the cells were incubated in donkey anti-goat immunoglobulin G-fluoresceinisothiocyanate (1:400, sc-2024; Santa Cruz) for 1 h at 4°C. Finally, cells were rinsed in PBS, coverslipped and examined with a confocal microscope (C1 si, Nikon, Japan).

### Western Blot analysis

Whole cell lysates were prepared from treated cells for Western blot analysis as described previously [37]. Before immunoblotting, the protein concentrations were determined with a BCA detection kit (Pierce, USA) and adjusted to equal concentrations across different samples. The proteins were separated by 10–12% SDS-PAGE gel and transferred to nitrocellulose membranes. The membrane was subjected to immunoblotting using enhanced chemiluminescence (ECL kit; Amersham Pharmacia Biotech, Piscataway, NJ, USA) and visualized by autoradiography. Protein β-actin (1:5000; A5441; Sigma) was used as the loading controls.

### Statistical Analysis

All of experiments were repeated at least three times. The data were presented as means ± SD. Statistical analysis was carried out by ANOVA followed by a Dunnett t-test, considering *P < 0.05 as significance. Statistical significance was assessed using GraphPad Prism 4 software.

## Results

### Res induces apoptosis of U251 cells

**Figure 1:**
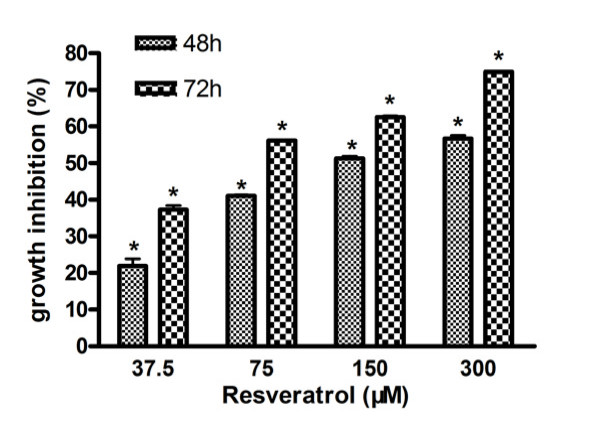
**Effects of Res on viability of U251 cells**. U251 cells following exposure to various concentrations of Res at different lengths of time and cell viability, analyzed with MTT assay. Values were given as mean ± SD; *, *p *< 0.05 vs control (*n *= 6).

U251 cells were treated with Res at concentrations ranging from 0 to 300 μM for various lengths of time. The cell viability of U251 cells was determined using the MTT assay. As shown in Figure. 1, Res reduced U251 cell viability in a time- and dose-dependent manner. After 48 h of treatment, the inhibitory rate of Res (150 μM) on viability of U251 cells had reached 51.29 ± 0.64%, and when the incubation time was prolonged to 72 h, the inhibitory rate increased to 62.56 ± 0.36%. While at the dose of 75 μM the inhibitory ratio was 41.03 ± 0.32% with 48 h of treatment. Based on this dose-effect relationship, Res concentration of 150 μM was used for subsequent studies.

**Figure 2:**
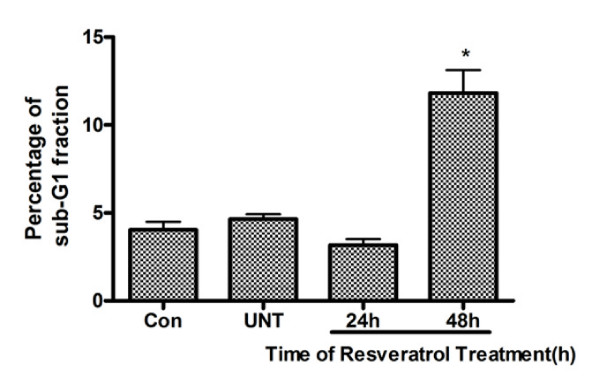
**DNA flow-cytometric analysis of U251 cells after treatment with Res**. The cells at the sub-G_1 _fraction were determined by DNA flow-cytometric analysis. Differences between control, UNT and 150 μM of Res treatment for 24 h or 48 h were observed; *, *p *< 0.001 (*n *= 6); data are presented as means ± SD.

To examine whether Res affects cell cycle and induces apoptosis, we performed the DNA flow cytometric analysis. As shown in Figure. 2, cells treated with Res (150 μM) for 48 h showed increased population of cells in sub-G1 phase, characteristic of apoptosis, from 4.04 ± 0.46% to 11.82 ± 1.30%.

**Figure 3:**
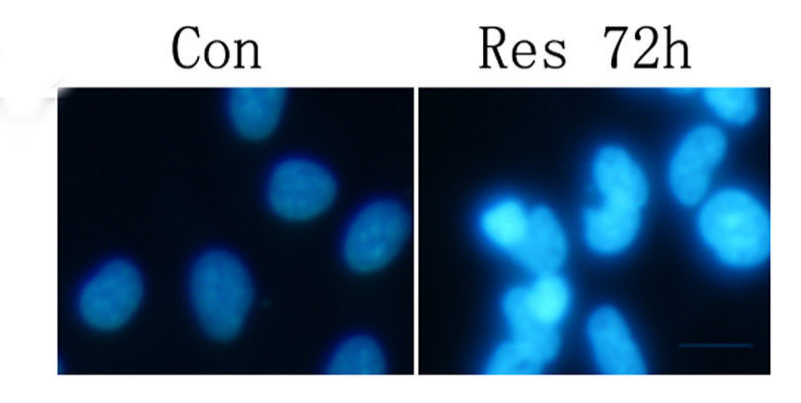
**Hoechst 33258 staining**. Nuclei of U251 cells were treated with Res (150 μM) for 72 h and stained with Hoechst 33258 to detect apoptosis morphologically. Microphotographs were shown as representative results from three independent experiments. ×400. Bar, 50 μm.

To determine the incidence of apoptosis morphologically, we stained the nuclei of treated U251 cells with Hoechst 33258. As shown in Figure. 3, apoptotic morphological characteristics such as chromatin condensation and nuclear fragmentation were detected in U251 cells treated with Res.

It has been suggested that Res-induced apoptotic death may be mediated by mitochondria-dependent signaling pathways [22]. Chemically promoted apoptosis mediated by the mitochondria/caspase-9 activation pathway is often, though not always, associated with the collapse of ΔΨm as a result of leakiness of the inner mitochondrial membrane [38]. Therefore, to investigate whether mitochondrial membrane integrity is damaged by Res, mitochondrial membrane potential was measured using JC-1, a sensitive fluorescent probe for ΔΨm [29].

**Figure 4:**
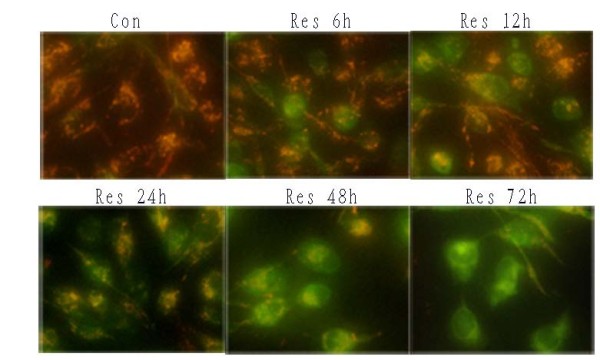
**Collapse of mitochondrial ΔΨm induced by Res**. U251 cells were cultured and incubated with Res (150 μM) for 6–72 h. Cells were stained with JC-1 and examined with fluorescence microscopy. Cells with normal mitochondria predominantly show red fluorescence. Cells with decreased mitochondrial membrane potential emit diffuse green fluorescence in the cytoplasm. Microphotographs were shown as representative results from three independent experiments. ×400.

U251 cells were cultured in 24-well plates and treated with Res for various lengths of time. When ΔΨm was low, JC-1 existed mainly in a monomeric form, which emits green fluorescence. As shown in Figure. 4, an increase in the amount of mitochondria with collapsed membrane potential was detected as early as 6 h after Res treatment.

**Figure 5:**
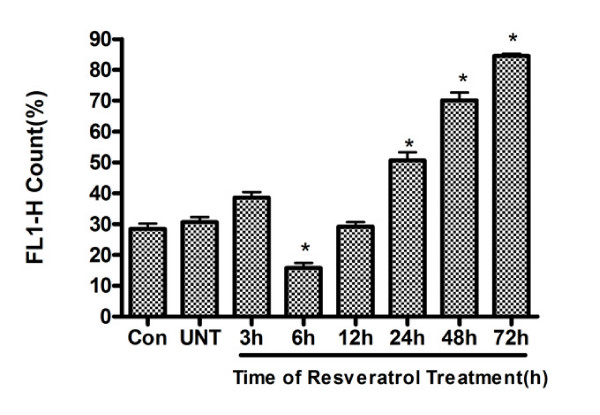
**Effects of Res on alterations in mitochondria membrane potential**. U251 cells were treated with Res (150 μM) for various lengths of time. Mean percentage of U251 cells stained green (FL 1-H) of all total cells was determined by flow cytometry. Results (mean ± SD) from 3 independent experiments were quantitatively analyzed. *, *P *< 0.05 compared to the control group.

To make a quantitative analysis, we use a flow cytometer (EPICS-XL, Beckman, USA). As shown in the Figure. 5, the levels of FL1 (green) count were found to be increased 6 h after Res treatment. This alteration reached its peak 72 h after Res treatment. These results indicate that a dysfunction of ΔΨm is an early event in Res-induced cell death.

**Figure 6:**
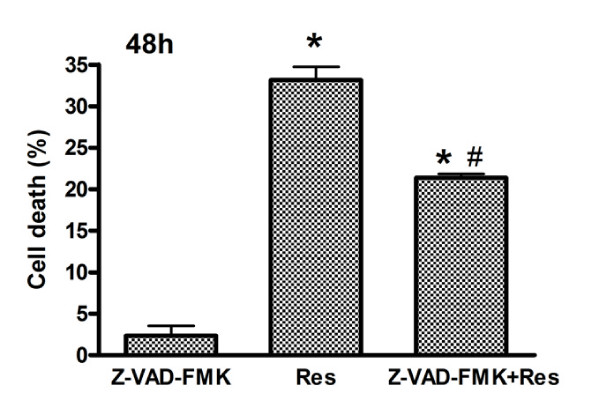
**Effect of Res on induction of apoptosis**. Effects of caspase inhibitors on Res-induced death of U251 cells. U251 cells were pretreated with Z-VAD-FMK 1 h before Res (150 μM). Cell viability was determined by MTT assay 48 h later. Data are presented as means; bars, ± SD.(*n *= 3). *, *p *< 0.05 compared to control group; #, *p *< 0.05 compared to Res alone within the treated group.

To confirm an apoptotic mechanism in Res-induced death of U251 cells, the effects of pretreatment with the pan-caspase inhibitor Z-VAD-fmk were investigated. U251 cells were pretreated with Z-VAD-fmk 1 h before Res treatment, cell viability was determined with MTT assay 48 h later. As shown in Figure. 6, Z-VAD-fmk significantly inhibited the cytotoxicity of Res.

### Res activates autophagy in U251 cells

**Figure 7:**
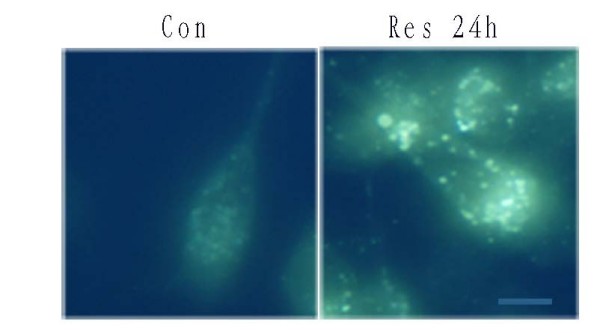
**MDC-labeled vesicles were induced after Res treatment**. U251 cells were incubated with Res(150 μM) for the indicated time and stained with MDC(50 μM). Fluorescence particles in the cytoplasm indicate autophagic vacuoles. Microphotographs were shown as representative results from 3 independent experiments. Magnification ×400. Bar, 50 μm.

As Res-treated U251 cells triggered a slow process of apoptosis, we next assessed whether Res induces autophagy in U251 cells. MDC accumulates in mature autophagic vacuoles, such as autophagolysosomes, but not in the early endosome compartment [33]. MDC staining can be used to detect autophagic vacuoles. When cells are viewed with a fluorescence microscope, AVs stained by MDC appear as distinct dot-like structures distributed within the cytoplasm or localizing in the perinuclear regions. As shown in Figure. 7, there was an increase in the number of MDC-labeled vesicles at 24 h after Res treatment. The result indicates an induction of AV formation by Res.

**Figure 8:**
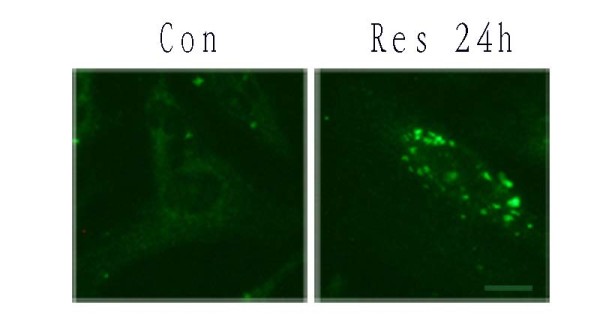
**LC3 immunofluorescence in U251 cells after Res treatment**. U251 cells were incubated with Res(150 μM) for the indicated time and stained with anti-LC3 antibody. Cells were examined by fluorescence confocal microscopy. Magnification ×400. Bar, 50 μm.

LC3 is localized in autophagosome membranes during amino acid starvation-induced autophagy [35,36]. LC3 is considered a molecular marker of autophagosomes. To assess if LC3 is altered in Res-induced autophagy, we examined localization of LC3 immunoreactivity. Under a fluorescence microscope, punctate patterns of LC3 immunoreactivity in many cells were observed after Res (150 μM) treatment, representing increased formation of autophagic vacuoles (Figure. 8). In contrast, the control cells only showed diffuse distribution of LC3 immunoreactivity.

**Figure 9:**
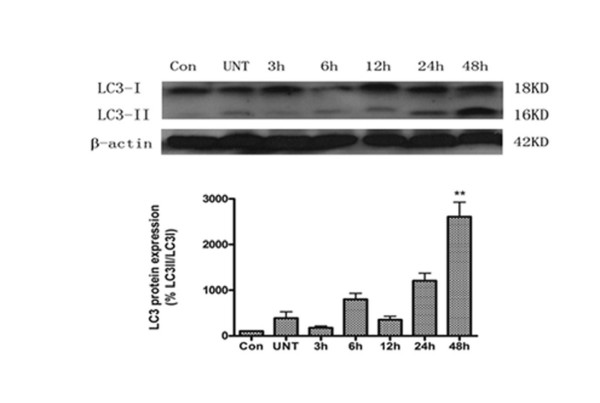
**LC3 protein expression in U251 cells**. Western blot analysis of LC3 expression after 3, 6, 12, 24, and 48 h of treatment with 150 μM of Res. **, *P *< 0.05 compared to the control group.

Recent investigations suggest that there are two forms of the LC3 proteins in various cells: LC3-I and LC3-II [36]. LC3-I is the cytoplasmic form and is processed into LC3-II, which is autophagosome membrane bound. Therefore, the amount of LC3-II is correlated with the extent of autophagosome formation. Using Western Blot analysis with anti-LC3 antibody, we examined the expressions of LC3-I (18 kDa) and LC3-II (16 kDa) in U251 cells after treatment with Res (150 μM). As seen in Figure. 9, an apparent increase in the levels of LC3-II protein was detected in U251 cells 24 h after treatment with Res, with a peak effect occurred at 48 h.

**Figure 10:**
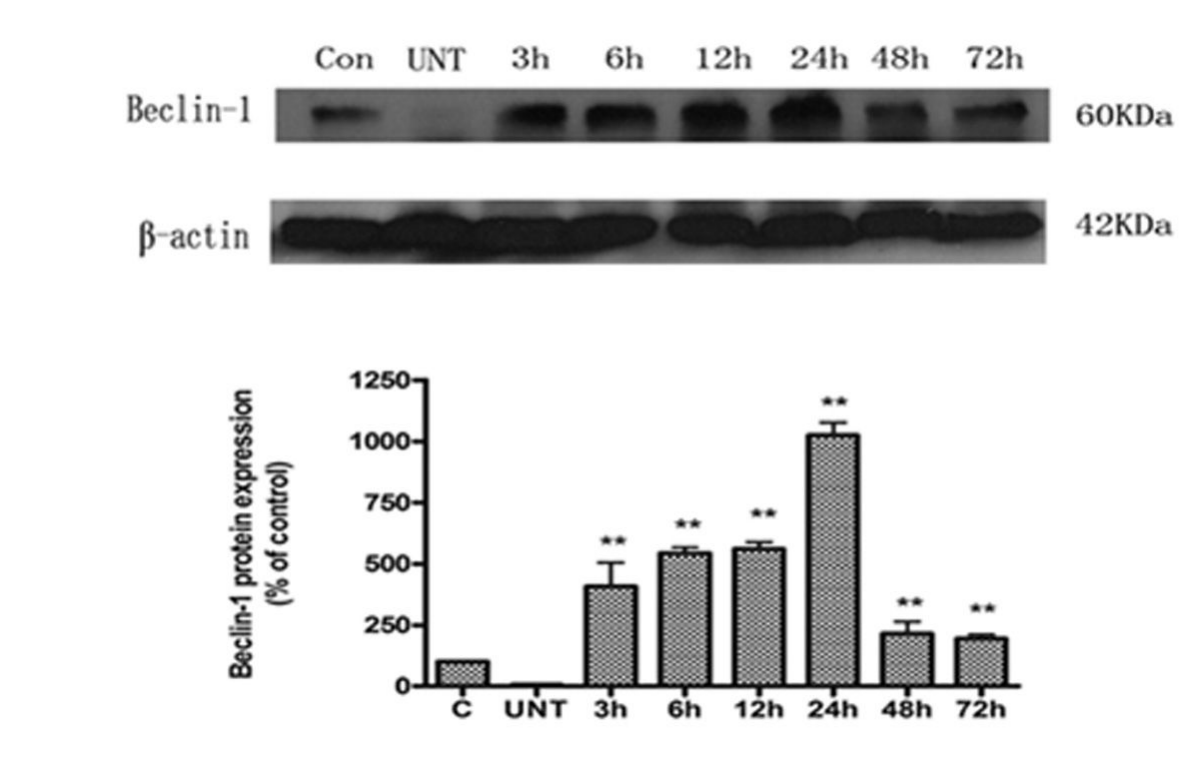
**Beclin-1 protein expression in U251 cells**. Western blot analysis of beclin-1 expression after 3, 6, 12, 24, 48, and 72 h of treatment with 150 μM of Res. **, *P *< 0.05 compared to the control group.

Autophagy is controlled by a group of evolutionarily conserved genes (ATG genes) [39,40]. More than 30 ATG genes have been identified in yeast and at least 11 have orthologs in mammals, Atg6 is known as beclin1. Beclin 1/Atg6 is a part of type III PI3 kinase complex that is required for the formation of the autophagic vesicle and interference with beclin 1 can prevent autophagy induction. Therefore, beclin 1 plays considerable roles in the processes of autophagy. The Western blot analysis revealed that beclin 1 levels were markedly increased 3 h after Res treatment, but decreased dramatically 24 h later (Figure. 10).

### Autophagy reduces Res-induced death of U251 cells

**Figure 11:**
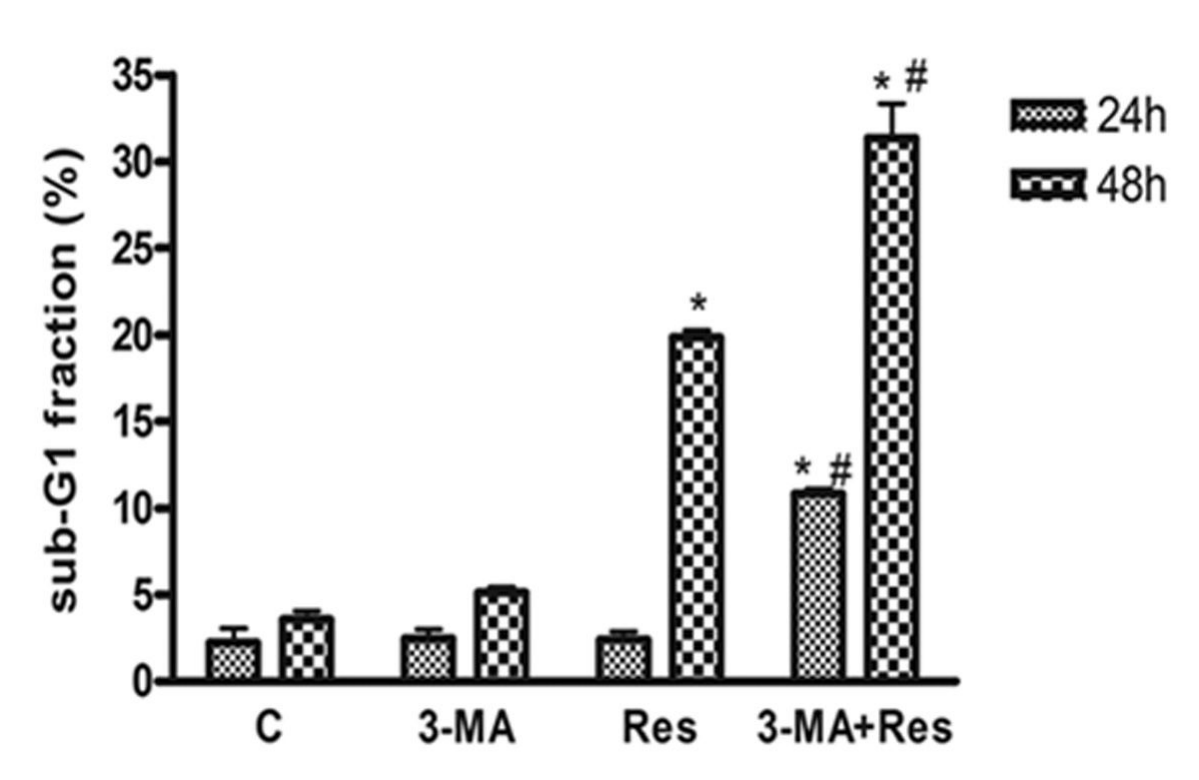
**Effects of 3-MA on Res-induced death of U251 cells**. U251 cells were pretreated with various doses of bafilomycin A1 1 h before Res (150 μM). The appearance of sub-G_1 _fraction was determined by DNA flow-cytometric analysis. Differences between control, Res (150 μM) and 3-MA (10 mM)+Res (150 μM) treatment for 24 or 48 h were observed. Data are presented as means ± SD (*n *= 6). *, *p *< 0.001 compared to control group; #, *p *< 0.001 compared to Res alone group.

3-MA, the specific class III PI3 kinase inhibitor, prevents autophagy at an early stage; while bafilomycin A1, a specific inhibitor of vacuolar H^+^-ATPase, prevents autophagy at a late stage by inhibiting fusion between autophagosomes and lysosomes. To investigate the effects of autophagy on Res-induced apoptosis, 3-MA was added 1 h before Res. Results showed that 3-MA alone had little effect on sub-G1 fraction, an indicator of apoptotic cell death; however, pretreatment of U251 cells with 3-MA significantly increased the sub-G1 fraction induced by Res (Figure. 11).

**Figure 12:**
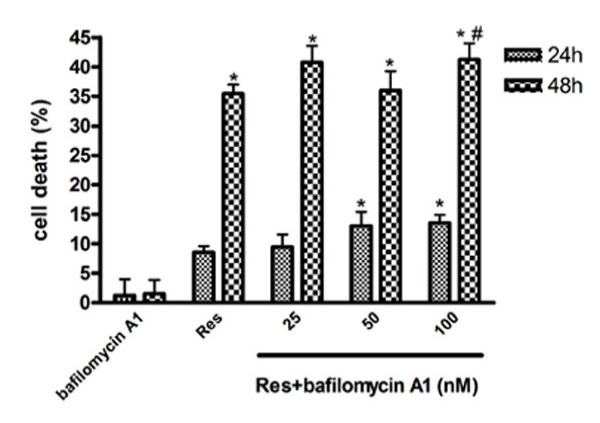
**Effects of bafilomycin A1 on Res-induced death of U251 cells**. U251 cells were pretreated with various doses of bafilomycin A1 1 h before Res (150 μM). Cell viability was evaluated by MTT assay 24 and 48 h later. Data are presented as means ± SD (*n *= 3). *, *p *< 0.05 compared to control group; #, *p *< 0.05 compared to Res alone group.

In addition, to explore inhibition of autophagy at a late stage, bafilomycin A1 was added before Res. As shown in Figure. 12, the specific inhibitor of vacuolar H^+^-ATPase bafilomycin A1 alone had no significant effect in U251 cells, however, Res-induced cytotoxicity was significantly potentiated by bafilomycin A1.

## Discussion

compounds used in cancer chemotherapy are derived from plant sources. Among them is hydroxylated stilbene Res, which is frequently found in many dietary products such as grapes, peanuts, berries and wine [41]. Previous study indentified the cancer chemopreventive activity of Res, which inhibits cellular events associated with tumor initiation, promotion and progression, more consistent with the actions of conventional chemotherapeutic drugs[] veratrol induces growth inhibition, S-phase arrest, apoptosis, and changes in biosion in several human cancer cell lines [42]. It has been shown that Res affects many different signal pathways in many types of cells. However, except for some preliminary reports, there is little experimental evidence supporting its efficacy in treating tumor cells or malignancies in animals [43,44]. Malignant gliomas are the primary brain tumor among many tumors. Previous study described that resveratrol induced apoptotic death in human U251 glioma cells [22]. In this study, we observed dose- and time-dependent cytotoxic effects of Res against U251 cells. These results are consistent with other reports showing the cytotoxic effects of Res to tumor cells [25,42].

Our present study suggests that Res induces autophagy in human U251 glioma cells. It was found that after Res treatment, vacuoles labeled by MDC increased in the cytoplasm. We also observed green punctate immunostaining of LC3 scattered in the cytoplasm. It has been demonstrated recently that LC3 is the first mammalian protein that is specifically recruited to autophagosome membranes [35]. These findings are similar to those showing autophagy activation after amino acid deprivation.

Whether autophagy promotes cell death or inhibits cell death is circumstantial. Some studies suggested that autophagy is a cell death mechanism. Autophagy is referred to as programmed cell death type II, while apoptosis is well known as programmed cell death type I [45,46]. Cells undergoing autophagic cell death or apoptosis display distinct morphological features. In apoptosis, there is condensation and fragmentation of DNA but preservation of organelles until late in the process. In contrast, in autophagic, or programmed cell death type II, there is early degradation of organelles whereas preservation of nucleus until late stages [47]. On the other hand, autophagy may serve as a key mechanism of cell survival[48]. This function of autophagy is an evolutionarily ancient process, conserved from yeast to mammals, and best characterized in nutrient deficiency. Autophagy can protect cells by inhibiting them from undergoing apoptosis[49]. In our study, inhibition of autophagy with 3-MA significantly increased the sub-G1 fraction and subsequently promoted U251 cells to cell death. Another specific inhibitor of the lysosomal protein pump ATP, bafilomycin A1, also increased Res-induced cytotoxicity. These findings suggest that autophagy plays an important role in Res-induced cell death and that inhibition of autophagy significantly affects the anti-tumor effects of Res. Autophagy may play an inhibitory role in the apoptotic process in U251 cell after treatment with Res. Therefore, agents that inhibit autophagy may be potential candidates to combine with Res for anti-tumor treatment.

Cell death is often associated with the collapse of ΔΨm [50]. The mitochondrion may integrate cell death signals and autophagy activation. Previous study suggested that apoptosis and autophagic cell death are interconnected through mitochondrial permeability transition(MPT) [51]. It has been shown that Res-induced apoptosis requires the activation of caspase-3 and increased release of cytochrome c. Accordingly, we observed a rapid decrease in ΔΨm after resveratrol treatment at an early stage. As a result, the loss of mitochondrial membrane polarization seem to be a intracellular signal that may be responsible for the induction of autophagy and apoptosis. Thus, the relationship among MPT, apoptosis and autophagy remains unclear, which requires further investigation.

## Conclusion

In summary, this study showed that Res induces autophagy in human U251 glioma cells. Autophagy and apoptosis play different roles in Res-induced death of U251 cells. Apoptosis is an important cause of death of U251 cells, while autophagy delays apoptosis and protect cells from death. These findings add a novel concept to Res-induced cell death pathways and suggest that autophagy inhibitors may be potential agents to enhance Res's anti-tumor efficacy.

## Competing interests

The authors declare that they have no competing interests.

## Authors' contributions

JL carried out all the experiments, performed the statistical analysis and drafted the manuscript; ZQ participated in revising the manuscript critically for important intellectual content; ZL participated in the design of the study and gave final approval of the version to be published. All authors read and approved the final manuscript.

## Pre-publication history

The pre-publication history for this paper can be accessed here:

http://www.biomedcentral.com/1471-2407/9/215/prepub
